# Expression of *AmGR10* of the Gustatory Receptor Family in Honey Bee Is Correlated with Nursing Behavior

**DOI:** 10.1371/journal.pone.0142917

**Published:** 2015-11-20

**Authors:** Yisilahaiti Paerhati, Shinichi Ishiguro, Risa Ueda-Matsuo, Ping Yang, Tetsuro Yamashita, Kikukatsu Ito, Hideaki Maekawa, Hiroko Tani, Koichi Suzuki

**Affiliations:** 1 The United Graduate School of Agricultural Sciences, Iwate University, Morioka, Iwate, Japan; 2 Organization for Research Promotion, Iwate University, Morioka, Iwate, Japan; 3 Biochemical Science Association, Shinagawa-ku, Tokyo, Japan; 4 Institute for Bee Products and Health Science, Yamada Apiculture Center, Inc., Kagamino, Okayama, Japan; AgroParisTech, FRANCE

## Abstract

We investigated the association between the expression of a gene encoding gustatory receptor (G10) and division of labor in the honey bee, *Apis mellifera*. Among 10 GR genes encoding proteins 15% ~ 99% amino acid identity in the honey bee, we found that *AmGR10* with 99% identity is involved in nursing or brood care. Expression of *AmGR10* was restricted to organs of the hypopharyngeal gland, brain, and ovary in the nurse bee phase. Members of an extended nursing caste under natural conditions continued to express this gene. RNAi knockdown of *AmGR10* accelerated the transition to foraging. Our findings demonstrate that this one gene has profound effects on the division of labor associated with the development and physiology of honeybee society.

## Introduction

Genetic and epigenetic studies can be fruitful in revealing the social structures of animal species. Diverse social organisms such as insects, fishes, voles, and humans have been used to explore the relations between genes, brains, and social behavior [1–3]. Conserved genes are crucial to understanding a wide range of characteristics from social cognition to clinical disorders; for example, *FoxP1* and *FoxP2* expression patterns in human fetal brain are similar to those in the songbird [4, 5]. Although some genes influence complex behaviors via pleiotrophic effects, manipulation of a single gene or its orthologs can have remarkable effects on behavior [1]; for example, disruption of a single copy of *FoxP2* in mice causes modest development delay and a significant alteration in ultrasonic vocalization [6]. Behavioral transitions in *Drosophila melanogaster* food searching behavior from rover to sitter [7] and in adult worker honey bees (*Apis mellifera*) from hive tasks to foraging [8] are caused by expression of the same foraging gene, *for*.

The honey bee is an important model organism in studies of nursing or brooding behavior. During the first 2–3 weeks of their adult lives, worker bees perform different tasks in the hive, including nursing or caring for larvae and the queen. After that, for remaining 5–7 weeks of their lives, they become foragers, collecting pollen and nectar [9–11]. This transition in behavior involves changes in the physiological processes of hormones and neurochemicals and in the expression of thousands of genes [12–14].

Although exposure to brood pheromone can delay onset of foraging and regulate the expression of genes in the brain [15], and *vitellogenin* influences social foraging specialization [16], a single nursing or brood care–related gene which causes an adverse effect to the foraging gene, may also be involved in the division of labor. Therefore, we looked for nursing-related genes that influence nursing behavior, in contrast with foraging behavior.

## Materials and Methods

### Bees

European honeybees were maintained in the hives 1 and 2 at Iwate University and in the hive 3 at the Tropical Biosphere Research Center, University of the Ryukyus. Newly emerged workers were marked with a small spot of enamel paint on the thorax for later identification [9]. Marked nurse bees (seen with their heads in larval cells) aged 0 to 14 days after adult emergence were collected in the hive (hive 1). Marked forager bees (carrying pollen or nectar) aged 19 to 29 days were collected on their return to the hive (hive 2) [9, 16]. An extended-duration nursing caste in 29-day-old adults within the hive (hive 2) was created under natural conditions during October to early November of 2007 [11]. We marked them and also used as the extended-duration nursing caste.

### Total RNA extraction and mRNA purification

To investigate the influence of gene action on behavior, we chose the hypopharyngeal gland (HPG), which initially synthesizes and secretes royal jelly, and later shrinks in foragers and synthesizes digestion enzymes, indicating a transition in an organ-level trait that reflects the division of labor [17]. To examine organ-specific expression, we also analyzed ovaries, brains, midguts, mandibular glands, and salivary glands.

Bees were anesthetized in insect saline (0.75% NaCl) on ice, and the organs were dissected out a binocular microscope. Total RNAs from bulked organs were prepared with Isogen (Nippon Gene) and frozen in liquid nitrogen and stored at –80°C until use. Polyadenylated RNA was separated with the aid of oligotex-dT30-coated magnetic beads (TaKaRa Bio), according to the manufacturer’s instructions.

### Differential display and subcloning, sequencing, construction of cDNA library, and PCR

These materials in experiments 1 and 2 were performed as described in supporting information.

### RT-PCR and cloning of *AmGR10* in nurse bees

For RT-PCR, the full-length cDNAs of 7-day-old nurse bees revers-transcribed using *Ex–Taq* polymerase (TaKaRa Bio) from *AmGR10* mRNA were amplified using from primers (forward, 5′–ATGATAGAACTCTCTAAGGC–3′; reverse, 5′–CGTACTTGGTGATCCTTACT–3′) obtained from the NCBI Honey Bee Genome Resources. PCR conditions were 9 min denaturation at 95°C; 35 cycles of 1 min at 94°C, 1 min annealing at step-down temperatures (72°C×3, 68°C×3, 64°C×3, 60°C×3, 56°C×3, 52°C×20), and 1 min extension at 72°C; and a final 7 min at 72°C. PCR products were separated in agarose gels, excised, purified with a DNA fragment purification kit (Toyobo), subcloned into the pCR 2.1 vector (Invitrogen), and cloned into INFαF′ cells (Invitrogen). The RACE products were sequenced as described in supporting information.

### Life-stage-specific RT-PCR of *AmGR10* in organs

To examine stage-specific expression during from egg to adult forager and the organ-specific distribution of *AmGR10* transcripts, we collected randomly 300 eggs within 24 h after oviposition, and 50 larvae and 10 pupae within the hive (hive 2) between June and October of 2007 and 2008. Total RNAs from these samples were frozen in liquid nitrogen and stored at –80°C until use. We subjected all RNA samples to first-strand reverse transcription. The reverse-transcribed cDNA samples were amplified with *AmGR10* primers. Expression was normalized to that of the constitutively expressed *β*–actin gene of *A*. *mellifera*, and was confirmed by RT-PCR [18].

### Preparation of double-stranded RNA

To synthesize double-stranded RNA (dsRNA) for RNA interference, we used a PCR-template method. Forward and reverse primer sequences to amplify a 513- base- pair region were selected from the *AmGR10* nucleotide sequence and from *A*. *mellifera* putative GR 10 from the NCBI Honey Bee Genome Resources. A T7-promoter sequence (TAATACGACTCACTCACTATAGGGAGA) was attached to the 5′ end of each primer to facilitate *in vitro* transcription of sense and antisense RNAs simultaneously. The following primers were used: forward, primer–5′–TAATACGACTCACTATAGGGAGACCACATAGAACTCTCTAAGGC–3′; reverse, primer–5′–TAATACGACTCACTATAGGGAGACCACAGTAAGGATCACCAAG–3′. PCR was performed with 100 ng plasmid DNA template, 25 pmol of each T7-linked primer, 8 mM MgCl_2_, 10×PCR buffer, all four deoxynucleotides at 5 mM, and 2.5 U of Taq DNA polymerase in a 50 μL PCR reaction. PCR was performed as for cloning. Amplified products were purified by gel extraction with a Mag Extractor–PCR & Gel Clean up kit (Toyobo) and used as templates for *in vitro* transcription for dsRNA synthesis in a Megascript RNAi kit (Ambion). Transcription products were treated as instructed by the user manual and re-suspended in nuclease-free water.

### RNA interference (RNAi)

Seven-day-old nurse bees were divided into three groups. The first group was injected through the neck membrane with 8.2 μg/2.0 μL *AmGR10* dsRNA (ds *AmGR10* group, *n* = 100 total in 4 independent experiments), as described previously [19]. As a handling control, the second group (sham group, *n* = 80 total in 4 independent experiments) received a 2.0 μL of nuclease-free water [19, 20]. The third group were left untreated (*n* = 80 total in 4 independent experiments). All bees were tagged with paint to identify the treatment. Immediately after the injections, the bees were returned to the same hive (hive 1–2010, hive 2–2011 and 2012, hive 3–2009), which held approximately 5,000–10,000 worker bees of all age classes and an egg- laying queen. Following the transition to foraging (as indicated by the presence of marked bees outside of the hives), they no longer engaged in any within- hive-tasks [9]. The bees were observed daily for a total of 4.5 h (10:00–12:00 and 13:00–15:30) over 3 consecutive days.

### qRT-PCR

Bees were anesthetized on ice, and their HPGs were removed. RNA was extracted in Isogen buffer according to the manufacturer’s instructions and frozen in liquid nitrogen and stored at –80°C until use. After treatment with RAase-free DNase (Promega), 2 μg of RNA was reverse-transcribed with AMV reverse transcriptase XL (RNA LA PCR kit [AMV] v. 1.1, Takara). The first-strand cDNAs were then amplified by quantitative real-time PCR using primers specific for honeybee *AmGR10* (forward, 5′–TGTCGGCGAGCTTATTTTCT–3′; reverse, 5′–TCGAAAGCAGGAGAGGAAGA–3′). Values were normalized to those of a *β*–actin gene of *A*. *mellifera* (AB023025; forward, 5′–TGCCAACACTGTCCTTTCTG–3′; reverse, 5′–AGAATTGACCCACCAATCCA–3′) [18]. Sequences were amplified in a 7500 Real-Time PCR System (Applied Biosystems) using a One Step SYBR PrimeScript RT-PCR kit (Takara) under conditions of 94°C for 10 s, followed by 55 cycles of 95°C for 5 s, 60°C for 15 s, and 72°C for 30 s. Each sample was analyzed in 4 independent experiments.

### Data analysis

We compared performance within an experimental group by Wilcoxon’s signed–rank test, and between groups by Mann–Whitney *U*–test.

## Results and Discussion

First we compared the expression of HPG mRNAs in nurse and forager bees. One candidate band sequence that was strongly expressed in nurse bees was used in a BLAST search of GenBank and the NCBI Honey Bee Genome (S1 Fig). 5’ and 3′ RACE-PCR amplified a gene fragment of 543 bases encoding 181 amino acid residues (S2 Fig) that had 99% sequence identity to gustatory receptor (GR) 10 of *A*. *mellifera* and 50% to that of a parasitoid jewel wasp, *Nasonia vitripennis* (S3 Fig) [21, 22].

The insect GR family was identified in the *Drosophila melanogaster* genome and named for its expression in gustatory organs such as the mouthparts [23]. So far, candidates for 68 GRs encoded by 60 genes have been identified in *D*. *melanogaster* [24] and candidates for 76 GRs encoded by 52 genes have been found *in Anopheles gambiae* [25]. AmGR1 in the honeybee antennae was recently shown to function as a sweet receptor that responds to some sugars, and AmGR2 in the same organ may act as a co-receptor [26], but Robertson and Wanner [21] revealed a total of 10 genes encoding proteins with 15% to 99% amino acid identity to each other and proposed that these 10 GRs are very limited in terms of gustatory mechanism and capacity. Here, we propose that the GR10 protein is primarily involved in nursing or brood-caring behavior in honey bees.

**Fig 1:**
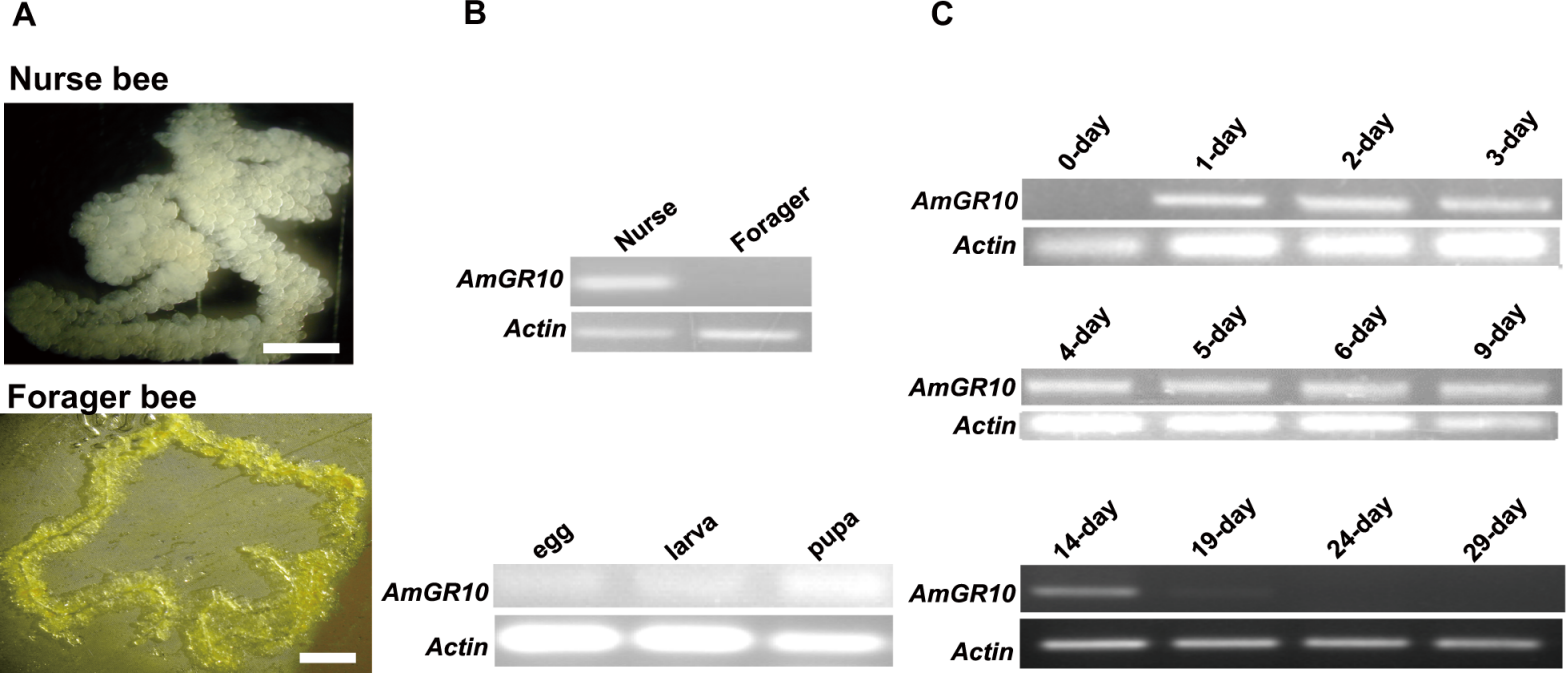
Expression of the *AmGR10* candidate in nurse and forager bees. (A) Photographs show HPGs from a nurse bee 7 days after adult emergence (upper) and from a forager bee collected randomly outside of the hive (hive 1) (lower). Scale bars = 0.5 mm. (B) RT-PCR analyses of *AmGR10* expression in nurse and forager bees and at each developmental stage. Upper gels show results from both adult castes in (A). Lower gels show results from 300 eggs within 24 h after oviposition, and 50 larvae and 10 pupae collected randomly between June and October of 2007 and 2008 (hive 1). HPGs taken from 20 nurse bees at 7 days after mergence and 20 forager bees collected randomly outside of the hive were dissected and pooled sample used for RT-PCR analyses. (C) RT-PCR analyses of *AmGR10* expression in HPGs during the transition between nurse and forager. HPGs were dissected from 20 workers of each indicated age (painted workers collected 0 to 14 days after mergence were identified as nurses, and those collected at 19, 24, and 29 days were identified as foragers) and pooled for RT-PCR analyses. We performed at least three RT-PCR analyses for each pooled sample.

We monitored the expression of *AmGR10* in the HPGs of 7-day-old nurse bees and 29-day-old foraging bees by using RT–PCR (Fig 1A). *AmGR10* was highly expressed in the HPGs of the nurse bees, but not of the foraging bees (Fig 1B). We then compared the expression of *AmGR10* in total RNAs prepared from newly laid eggs and in larvae and pupae collected randomly. *AmGR10* was not expressed in the eggs or larvae, but it was expressed at low levels in the pupae (Fig 1B). It was expressed in the HPGs of nurse bees from age 1 to 14 days, but not in foraging bees from age 19 to 29 days (Fig 1C). These expression profiles reflect the division of labor and physiological processes between nurse and foraging workers [1, 9, 10, 12].

**Fig 2:**
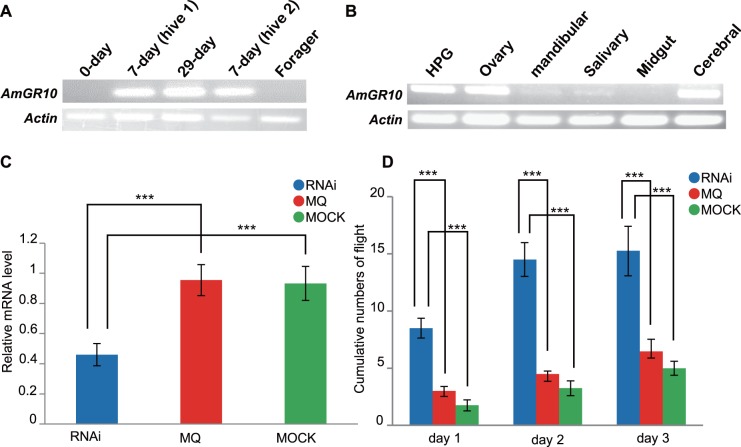
Expression and function of *AmGR10* in honeybees. (A) Artificial expression of *AmGR10* in the nursing state. In October 2007, we collected nurse bees 0 and 7 days old (the latter from hives 1 and 2). In early November, we collected 29-day-old workers in an extended nursing state within the hive and foragers outside of the hive at the same time (hive 2). Twenty HPGs from each group were pooled for RT-PCR analyses. (B) Organ distribution of *AmGR10* expression in 20 HPGs, 150 ovaries, 150 mandibular glands, 150 salivary glands, 20 midguts, and 20 cerebral brains from 7-day-old nurse bees between July and October of 2007 and 2008 (hive 2). Organ preparations from each group were pooled for RT-PCR analyses. (C) Knockdown of *AmGR10* mRNA in the HPGs of 7-day-old nurse bees. One hundred bees in 4 independent experiments (25 bees × 4) injected with *ds AmGR10* (8.2 μg⁄2 μL, “RNAi”), 80 bees in 4 independent experiments (20 bees × 4) injected with nuclease-free water (2 μL, “MQ”), and 80 untreated bees in 4 independent experiments (20 bees × 4) (“Mock”) were placed back into their hives (hives 1, 2, and 3) immediately after treatment and were collected again 24 h later for qRT-PCR. For qRT-PCR analyses, 3 bees were used in 4 independent experiments (3 bees × 4) and the remaining bees were allowed to forage. All values are means ± SEM. *** *P* < 0.001 compared with each control. (D) Effect of RNAi on foraging behavior. We ascertained the age in each group from paint markings: 22 bees × 4 in “RNAi”, 17 bees × 4 in “MQ”, and 17 bees × 4 in “Mock”. We observed the bees over 3 consecutive days outside of the same hive (see RNAi section in Materials and Methods) and their flights to ensure the onset of flight hazard were shown as cumulative numbers of bees initiating foraging, but thereafter we did not follow their survival or labors. All values are means ± SEM. *** *P* < 0.001 compared with each control.

Next, we created an artificially extended nurse state, in which the HPGs are hypertrophied and remain active [11]. *AmGR10* remained expressed even in 29-day-old nurse bees, unlike in foragers (Fig 2A). In nurse bees aged 7 days, we found *AmGR10* expression not only in the HPGs, but also in the brain and ovary (Fig 2B). Our results provide new evidence for a nurse-specific gene linked to the HPG, brain, and reproductive organs.

If *AmGR10* is fundamentally involved in worker behavior, its function should be detectable in the transition to foraging. To investigate this possibility, we generated *AmGR10*-knockdown bees by injecting ds RNA into 7-day-old nurse bees. These workers had significantly lower levels of *AmGR10* mRNA in the HPG than controls (Fig 2C). Monitoring of bee movements showed that this knockdown of *AmGR10* activity caused earlier nurse-to-forager transition (Fig 2D, S1 Video). Although the collective activities of foraging workers remain to be demonstrated [12], our data strongly support the notion that the *AmGR10* influences nursing behavior.

Complex relations between crucial genes and reversible DNA methylation have challenged our molecular understanding of the division of labor in honey bees [1, 3, 27], but little is known about how such genes or epigenetic changes can explain the complex pathways that determine the division of labor. Examination of the nine genes encoding GRs in *A*. *mellifera* by qRT-PCR has revealed that the expression of seven of them is enriched in gustatory organs such as the labial palps and the glossa, and *AmGr7* is expressed at high levels in the heads (although *AmGR10* was not investigated) [21]. Our finding of *AmGR10* as crucial to nursing or brood-caring behavior in the hive will help to explain how the GR protein family in social insects mediates the synthesis of royal jelly, as well as how it contributes to behavior in the hive. Although the ligand–receptor binding of the *AmGR10* product remains to be analyzed, our results suggest that *AmGR10* is important in the organization of honeybee societies.

In the invertebrate model system *D*. *melanogaster*, recent research has uncovered the fact that *GR43a* is expressed in a group of neurons in the posterior superior lateral protocerebrum; it is both necessary and sufficient to sense hemolymph fructose, and promote feeding in hungry flies but suppress feeding in satiated flies [28, 29]. Trehalose and glucose are the main hemolymph sugars in many insects, but fructose also is a main sugar in honey bee [30, 31]. This unique role of *Drosophila* GR43a in the sensing of fructose in the diet and the hemolymph may provide new insights into the mechanisms of the division of labor in honey bee and the nutrient receptor function of *AmGR10* in the HPG, brain, and ovary.

## Supporting Information

S1 Fig Differential expression patterns of genes by gel electrophoresis. Total RNA samples from 4- to 7-day-old nurse bees and foragers collected randomly outside of the hive were prescreened according to the Seegene user manual, and some differentially expressed genes were found. (A) In experiment 1, bands in columns GP 21, 23, 32, and 34 are differentially expressed genes between nurse bees and foragers (arrows). These bands were reamplified and directly sequenced (see Materials and Methods). Genes 1, 2, and 7 showed no significant similarity, but gene 6 showed high similarity to a royal-jelly-related milk protein (pRJP57-1) of *A*. *mellifera* [17]. (B) In experiment 2, one gene overexpressed in nurse bees (arrow) was subcloned (see S2 and S3 Figs). ACPs 11–20 are alternative primers.
(EPS)

S2 Fig Partial cDNA and deduced amino acid sequences of a gustatory gene *AMGR10* from *Apis mellifera*. *Terminal codon. A putative polyadenylation signal is boxed. *N*-glycosylation sites are underlined.
(EPS)

S3 Fig Comparison of amino acid sequences of gustatory proteins from honey bee and jewel wasp: gustatory receptor 10 (Nurs) in *Apis mellifera* identified from our experiments; gustatory receptor 10 (GR10) in *A*. *mellifera*; and GR10 in *Nasonia vitripennis*. Identical amino acids are shown in red. Dashes indicate gaps introduced to maximize sequence similarity.
(EPS)

S1 Materials and Methods Differential display and subcloning. **Amplication and PCR products were performed as described previously (**Yang P, et al., 2008).
(DOCX)

S2 Materials and Methods Sequencing. (DOCX)

S3 Materials and Methods Construction of cDNA library. (DOCX)

S4 Materials and Methods RACE. (DOCX)

S1 Video Effects of the injection of RNA interference on foraging onset. Seven-day-old nurse bees were injected through the neck membane with ds RNA (513-bp; ethanol-precipitated, suspended in nuclease-free water at 8.2 μg/2 μL) or with 2 μL of nuclease-free water (handling control), or were not treated (mock). The bees were observed daily for a total of 4.5 h (10:00–12:00 and 13:00–15:30) for 3 consecutive days, and the emergence of newly transitioned forager bees (tagged in white paint on the thorax), was recorded by video.
(AVI)
