# Supplementary material for: Expression of AmGR10 of the Gustatory Receptor Family in Honey Bee Is Correlated with Nursing Behavior
Source: PLoS One. 2015 Nov 20;10(11):e0142917. doi: 10.1371/journal.pone.0142917 (PMC4654511; doi:10.1371/journal.pone.0142917)
Supplement: S1 Materials and Methods — Amplication and PCR products were performed as described previously (Yang P, et al., 2008). (DOCX) [file pone.0142917.s004.docx]

**S1 Materials and Methods. This is the S1 Materials and Methods: Differential display and subcloning.**

HPGs of 10 bees (hive 2) anesthetized on ice were dissected in 0.75% NaCl. Total RNA was reverse-transcribed to cDNA with Super Script III reverse transcriptase (Invitrogen). Differential display was performed according to the Seegene Inc. user manual. Using the GeneFishing DEG kit, in experiment 1, we prescreened the samples and found differentially expressed genes (S1 Fig.). The bands were reamplified, extracted from the gel, and directly sequenced. In experiment 2, HPG-enriched cDNAs were subcloned into the pCR 2.1 vector (TA Cloning kit, Invitrogen) according to the manufacturer’s instructions and transformed into *Escherichia coli* INVαF′ cells. Amplification and PCR products were performed as described previously [1].

**References**

1. Yang P, Tanaka H, Kuwano E, Suzuki K. A novel cytochrome P450 gene (*CYP4G25*) of the silkmoth *Antheraea yamamai* : Cloning and expression pattern in pharate first instar larvae in relation to diapause. J. Insect Physiol. 2008; 54: 636 -643. doi: 10.1016/j.jinsphys.2008.01.001
