# Supplementary material for: Expression of AmGR10 of the Gustatory Receptor Family in Honey Bee Is Correlated with Nursing Behavior
Source: PLoS One. 2015 Nov 20;10(11):e0142917. doi: 10.1371/journal.pone.0142917 (PMC4654511; doi:10.1371/journal.pone.0142917)
Supplement: S2 Materials and Methods — (DOCX) [file pone.0142917.s005.docx]

**S2 Materials and Methods. This is the S2 Materials and Methods: Sequencing.**

The above plasmids containing inserts that hybridized with specific cDNA were purified with a QIAprep Spin Plasmid kit (Qiagen). The inserts were sequenced with a BigDye Terminator Cycle Sequencing kit (Applied Biosystems) on an ABI model 3100 sequencer. The sequences were used in BLAST searches of GenBank and the NCBI Honey Bee Genome.
