# Supplementary material for: Expression of AmGR10 of the Gustatory Receptor Family in Honey Bee Is Correlated with Nursing Behavior
Source: PLoS One. 2015 Nov 20;10(11):e0142917. doi: 10.1371/journal.pone.0142917 (PMC4654511; doi:10.1371/journal.pone.0142917)
Supplement: S3 Materials and Methods — (DOCX) [file pone.0142917.s006.docx]

**S3 Materials and Methods. This is the S3 Materials and Methods: Construction of cDNA library.**

A 3.5- µg sample of polyadenylated mRNA from 7-day-old nurse bees was used to prepare cDNA in a SMART PCR cDNA Synthesis kit (Clontech).
