# Supplementary material for: Expression of AmGR10 of the Gustatory Receptor Family in Honey Bee Is Correlated with Nursing Behavior
Source: PLoS One. 2015 Nov 20;10(11):e0142917. doi: 10.1371/journal.pone.0142917 (PMC4654511; doi:10.1371/journal.pone.0142917)
Supplement: S4 Materials and Methods — (DOCX) [file pone.0142917.s007.docx]

**S4 Materials and Methods. This is the S4 Materials and Methods: RACE.**

To identify the whole length of the *AmGR10* cDNA, we repeated 5′ and 3′ RACE-PCR with a SMART RACE cDNA Amplification kit (Clontech).
